# Thymic stromal lymphopoietin in human pancreatic ductal adenocarcinoma: expression and prognostic significance

**DOI:** 10.18632/oncotarget.25997

**Published:** 2018-08-28

**Authors:** Barbara Vizio, Monica Boita, Carmen Cristiano, Jasenka Mazibrada, Ornella Bosco, Anna Novarino, Adriana Prati, Savino Sciascia, Giovanni Rolla, Libero Ciuffreda, Giuseppe Montrucchio, Graziella Bellone

**Affiliations:** ^1^ Department of Medical Sciences, University of Turin, 10126 Turin, Italy; ^2^ Division of Allergy and Immunology, Department of Medical Science, Azienda Ospedaliera Ordine Mauriziano Umberto I, University of Turin, 10126 Turin, Italy; ^3^ Department of Medical Oncology, Azienda Ospedaliera Città della Salute e della Scienza di Torino, 10126 Turin, Italy; ^4^ Bradford Teaching Hospitals NHS Trust, Duckworth Ln, Bradford BD9 6RJ, United Kingdom; ^5^ Center of Research of Immunopathology and Rare Diseases-Coordinating Center of the Network for Rare Diseases of Piedmont and Aosta Valley, and SCDU Nephrology and Dialysis, S. Giovanni Bosco Hospital and University of Turin, 10154 Turin, Italy

**Keywords:** thymic stromal lymphopoietin, pancreatic ductal adenocarcinoma, T helper 2, OX40L, dendritic cells

## Abstract

Thymic stromal lymphopoietin (TSLP) has emerged as an important, but contradictory, player conditioning tumor growth. In certain contexts, by driving T helper (h) 2 responses via tumor-associated OX40 Ligand (OX40L)^+^ dendritic cells (DCs), TSLP may play a pro-tumorigenic role. The study elucidates the importance of TSPL in pancreatic ductal adenocarcinoma (PDAC), by analyzing: i) TSLP levels in PDAC cell-line supernatants and plasma from patients with locally-advanced/metastatic PDAC, pre- and post-treatment with different chemotherapeutic protocols, in comparison with healthy donors; ii) TSLP and OX40L expression in PDAC and normal pancreatic tissues, by immunohistochemistry; iii) OX40L expression on *ex vivo*-generated normal DCs in the presence of tumor-derived TSLP, by flow cytometry; iv) clinical relevance in terms of diagnostic and prognostic value and influence on treatment modality and response.

Some PDAC cell lines, such as BxPC-3, expressed both TSLP mRNA and protein. Normal DCs, generated *ex vivo* in the presence of TSLP-rich-cell supernatants, displayed increased expression of OX40L, reduced by the addition of a neutralizing anti-TSLP polyclonal antibody. OX40L^+^ cells were detected in pancreatic tumor inflammatory infiltrates. Abnormally elevated TSLP levels were detected in situ in tumor cells and, systemically, in locally-advanced/metastatic PDAC patients. Of the chemotherapeutic protocols applied, gemcitabine plus oxaliplatin (GEMOX) significantly increased circulating TSLP levels. Elevated plasma TSLP concentration was associated with shorter overall survival and increased risk of poor outcome. Plasma TSLP measurement successfully discriminated PDAC patients from healthy controls.

These data show that TSLP secreted by pancreatic cancer cells may directly impact PDAC biology and patient outcome.

## INTRODUCTION

Tumor-related inflammation frequently exhibits profiles that promote tumor growth and protect malignant cells from the immune system [1]. Cancer cells can create a favorable microenvironment by co-opting cells of the innate immune system, such as myeloid-derived suppressor cells, to become components of the pro-tumorigenic stroma [2]. They can also avoid the host’s tumor-specific T-cells by skewing the acquired immune response from cytotoxic T helper (h) 1 toward the permissive Th2 phenotype, and/or by inducing immunosuppressive regulatory T cells (T_reg_) [3].

Pancreatic adenocarcinoma (PDAC) is one of the deadliest epithelial cancers, because of the advanced stage at diagnosis, early systemic dissemination, and poor response to chemo/radio therapy. Clinical and experimental evidence support the occurrence of profound local and systemic immune-response alterations in PDAC patients [4–7].

Thymic stromal lymphopoietin (TSLP) was originally identified as a murine thymic stroma-secreted cytokine having regulatory functions on the growth and differentiation of B cells and the proliferation of T cells [8]. More recently, it was found to be highly expressed also outside the thymus, namely by epithelial cells of the lung, intestine and skin, and by fibroblasts, mast cells, and smooth muscle cells [9]. It is recognized as a critical mediator involved in the maintenance of Th2-type homeostasis at barrier surfaces [10]. In humans, TSLP contributes to the onset of allergic inflammatory diseases, by activating CD4^+^ T cells and inducing their Th2 differentiation [11]. This can occur either directly [12], or indirectly via the education of immature myeloid-derived DCs, the major TSLP-responsive cellular subset [13]. It is known that DCs play a critical role in polarizing Th effector cells, depending on their specific profile of cytokines and co-stimulatory molecules, and on the influence of the individual tissue milieu from which they originate. DCs treated *in vitro* with TSLP fail to produce the Th1-polarizing cytokine Interleukin (IL)-12, and up-regulate the expression of OX40 ligand (OX40L). This ligand is critical for its ability to polarize naïve T cells into inflammatory Th2 cells, producing Th2-type cytokines like IL-4, IL-5, IL-13, plus Tumor Necrosis Factor (TNF)-α [14].

For many types of cancers, including PDAC, a Th2 response predominates over the cytotoxicity induced by CD8^+^ T cells and the Th1 response. Generally, tumors with this phenotype have a worse prognosis than tumors in which Th1-type responses predominate [15]. However, the mechanism whereby Th2-biased immune responses are initiated in tumors remains poorly understood.

Recent studies in humans show that TSLP, being expressed in the tumor microenvironment, plays a role in promoting a Th2-like environment in the tumor. A study on pancreatic cancer, in which a Th2 (GATA3^+^) cellular infiltrate is predominant, identified a central role for cancer-associated fibroblasts (CAFs) in conditioning DCs with Th2-polarizing capability, via TSLP secretion [16]. *In vitro*, supernatants from CAF induced myeloid DCs to up-regulate the TSLP receptor (TSLPR), to secrete Th2-attracting chemokines, and to acquire a TSLP-dependent Th2-polarizing capability [16]. Importantly, *in vivo*, TSLPR-expressing DCs were found be present in the tumor stroma and in tumor-draining lymph nodes, but not in non-draining lymph nodes [16]. Moreover, this Th2 immune deviation plays an active role in tumor progression, and has been found to reduce patient survival [16]. However, the role of TSLP in cancer appears increasingly controversial, even paradoxical, as it exerts either pro- or anti-tumorigenic effects, depending on the context [17].

The current study sought to elucidate the importance of TSPL in PDAC development and progression, by analyzing expression of the cytokine *in vitro* and *in vivo*, and examining its clinical relevance in terms of diagnostic power, prognostic value, and influence on treatment modalities.

## RESULTS

### PDAC cell lines express TSLP at mRNA and protein levels

**Figure 1:**
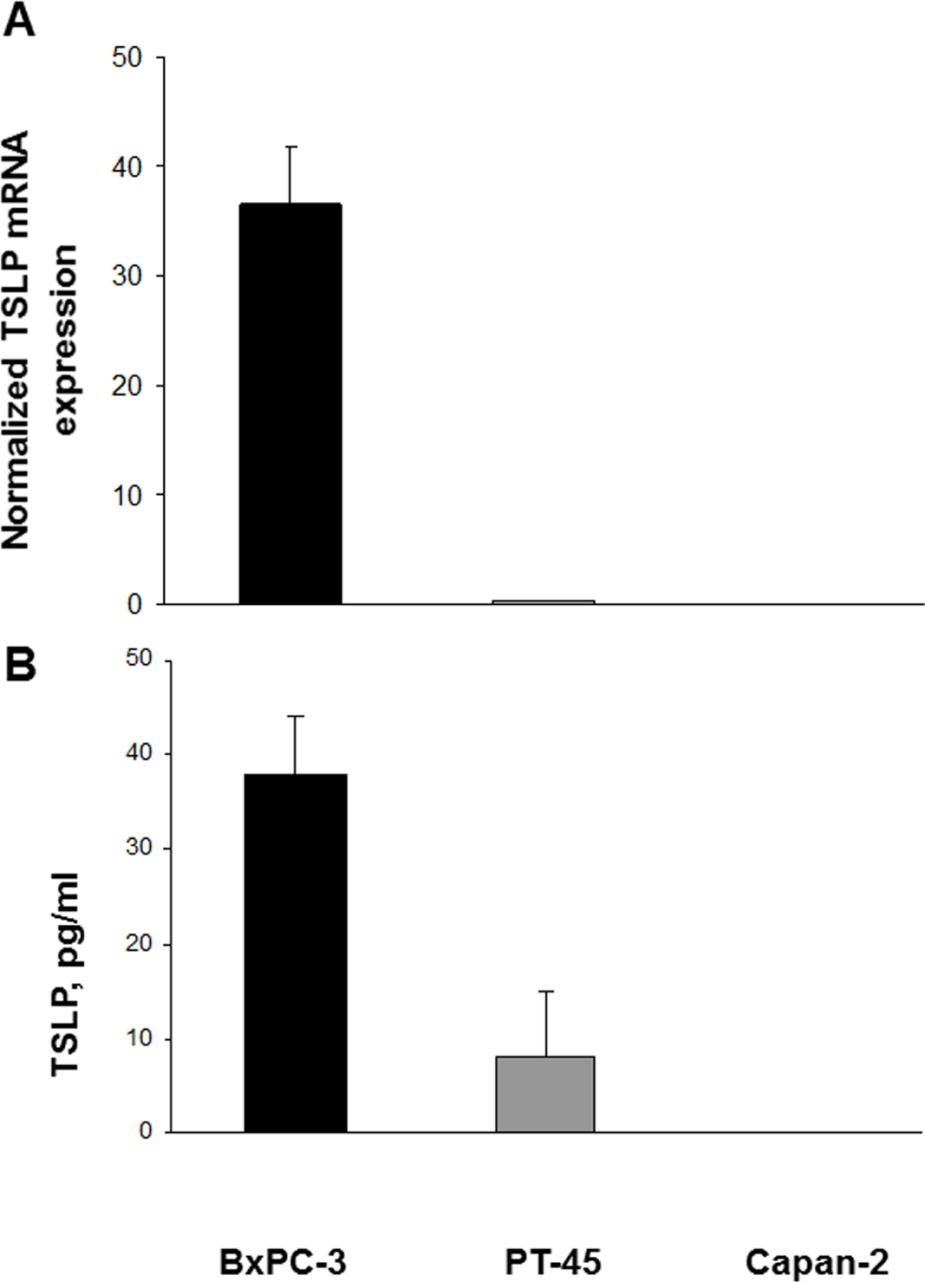
Expression of *TSLP* mRNA and protein in established pancreatic carcinoma cell lines (**A**) Cytokine mRNA levels in BxPC-3, PT-45 and Capan-2 cells were assessed by real time RT-PCR and normalized to β-actin mRNA levels. Values are means (±SD) normalized gene expression. (**B**) Concentration of TSLP in CM derived from BxPC-3, PT-45, and Capan-2 cells measured by ELISA. The mean level (±SD) of cytokine detected in triplicate in CM samples is indicated.

TSLP mRNA expression levels were first analyzed quantitatively in the established PDAC cell lines BxPC-3, PT-45, and Capan-2, using real time RT-PCR. As shown in Figure 1A, large amounts of TSLP transcript were found in BxPC-3 cells, minimal amounts in PT-45, and almost none in Capan-2 cells. To determine whether mRNA expression translates into protein production, TSLP levels were assessed in the conditioned medium (CM) of the three cell lines by specific ELISA. Figure 1B shows that, constitutively, BxPC-3 cells released 37.9 ± 6 pg/ml of TSLP, PT-45 cells were at the detection limit (8 ± 7 pg/ml), whereas Capan-2 cells were below the detection limit.

TSLP release from the non-tumorigenic immortalized human pancreatic ductal epithelial cells [HPDE6-E6E7 (H6c7)] was also checked; levels in the supernatant were undetectable (data not shown).

### TSLP expression in PDAC tissue samples

**Figure 2:**
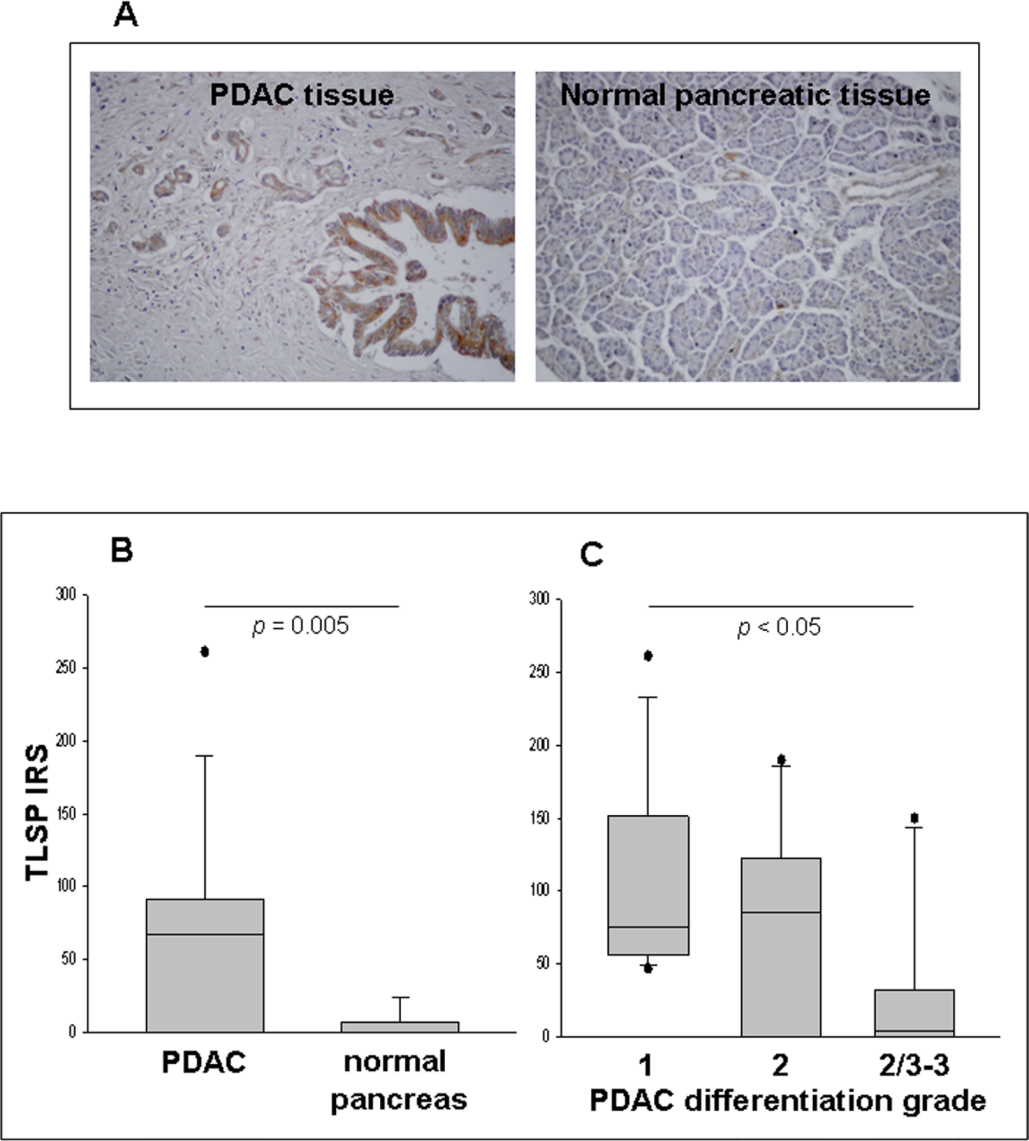
Detection of TSLP in PDAC and normal pancreatic tissues *in situ*, as determined by IHC (**A**) Immunostaining of representative samples of PDAC and normal pancreatic tissues (original magnification, ×200). (**B**) Immunoreactivity scores (IRS) in malignant (*n* = 38) and normal (*n* = 8) pancreatic tissue samples. *P*-values obtained by the Mann–Whitney Rank Sum Test. (**C**) IRS in PDAC categorized by tumor differentiation grading. *P*-values obtained by Kruskal–Wallis One Way Analysis of Variance by Ranks followed by Dunn’s Test. Median, 10th, 25th, 75th, and 90th percentiles are presented as vertical boxes with error bars. Dots indicate outliers.

*In situ* TSLP protein expression was then analyzed in malignant (*n* = 38) and normal (*n* = 8) pancreatic tissue specimens. Figure 2A shows representative examples of immunohistochemical staining, in PDAC and normal pancreas specimens. Seventy three % of PDAC cases expressed TSLP on both ductal and stromal cells, whereas only 25% of normal pancreas specimens did so. The semiquantitative assessment of staining (IRS), only evaluated on the ductal normal and malignant cells, demonstrated that TSLP levels were significantly higher in PDAC than in normal pancreas [IRS median (range): 61 (0–261) *vs.* 0 (0–24), *p* = 0.005] (Figure 2B). When PDAC cases were stratified by disease stage, there was no significant difference between groups (Supplementary Figure 1, *p* > 0.05). Conversely, when PDAC were classified by degree of tumor differentiation, well-differentiated tumors (grade 1) showed greater TSLP expression than did poorly-differentiated tumors (grades 2/3 plus 3) [IRS median (range): 75 (47–261), *vs.* 4 (0–150), respectively, *p* < 0.05], while moderately-differentiated tumors (grade 2) expressed similar TSLP levels both to well-differentiated tumors [IRS median (range) 85 (0–190) *vs.* 75 (47–261), *p >* 0.05] and poorly-differentiated tumors [IRS median (range) 85 (0–190) *vs.* 4 (0–150), *p* > 0.05] (Figure 2C).

### OX40L expression in normal DCs in the absence or presence of BxPC-3 or Capan-2 cell-CM

**Figure 3:**
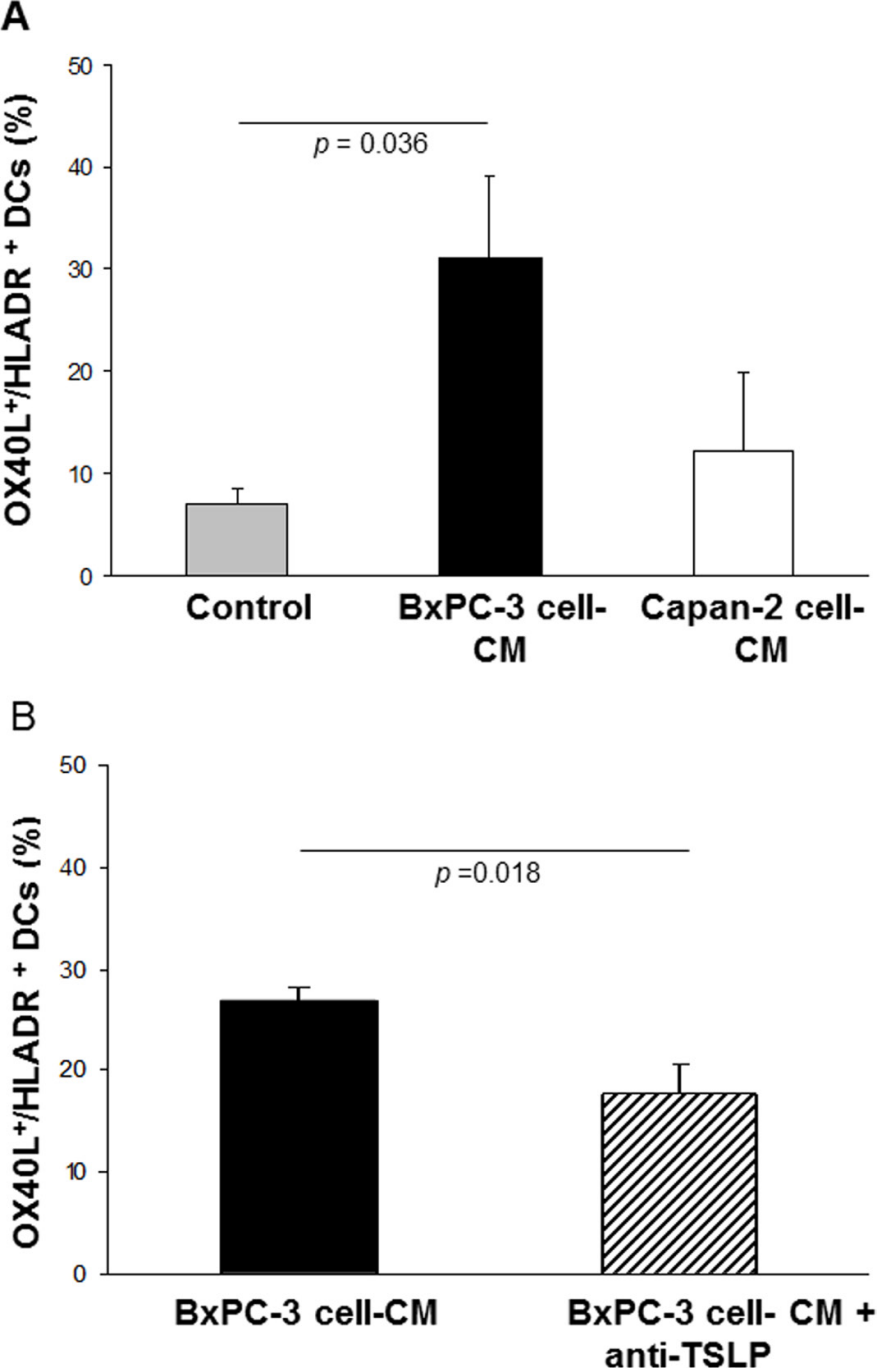
Effects of BxPC-3 and Capan-2 cell-CM on OX40L expression in *ex vivo*-generated normal DCs Incidence of OX40L expression on normal HLA-DR^+^ DCs obtained (**A**) in the absence (control), and in the presence of 20% BxPC-3 or Capan-2 cell-CM. Values are means ± SE. *P*-values obtained by One Way ANOVA followed by Bonferroni *t*-Test, or (**B**) in the presence of BxPC-3 cell-CM, pre-treated with a neutralizing anti-TSLP polyclonal antibody. Values are means ± SE. *P*-values obtained by the Paired *t*-Test.

Some PDAC cells can release biologically-significant amounts of TSLP, which can induce expression of OX40L in myeloid DCs. In turn, this leads to expansion of T cells producing type 2 cytokines, which are not very effective at contrasting tumor growth. The effect of CM of BxPC-3 and Capan-2 cells (respectively high and low TSLP producers) on the *ex vivo* differentiation of normal monocytes into DCs (*n* = 6) was thus assessed in terms of OX40L expression. As Figure 3A shows, only a small fraction of DCs generated in the absence of cell line CM expressed OX40L (mean % ± SE: 7.0 ± 1.3 HLA-DR^+^/OX40L^+^). Conversely, DCs differentiated in the presence of BxPC-3 cell-CM displayed a significant rise in OX40L expression *vs.* untreated DCs (mean % ± SE: 31.0 ± 8.0 HLA-DR^+^/OX40L^+^, *p* = 0.036). The CM of Capan-2 cell line, which produces TSLP in trace amounts, induced a slight but not statistically-significant increase in OX40L expression in DCs compared to DCs control (mean % ± SE: 12.2 ± 7.7 HLA-DR^+^/OX40L^+^, *p* = 0.497). The expression of OX40L on the surface of DCs generated in the presence of BxPC-3 cell-CM was partially, but significantly, reduced (34%) by the use of a neutralizing anti-TSLP polyclonal antibody with proven blocking efficacy (BxPC-3 cell-CM plus anti-TSLP DCs: mean % ± SE 17.8 ± 1.6 *vs*. BxPC-3 cell-CM DCs: mean % ± SE 26.8 ± 2.8, *p* = 0.018) (Figure 3B and Supplementary Table 1).

### OX40L expression in PDAC tissue samples

**Figure 4:**
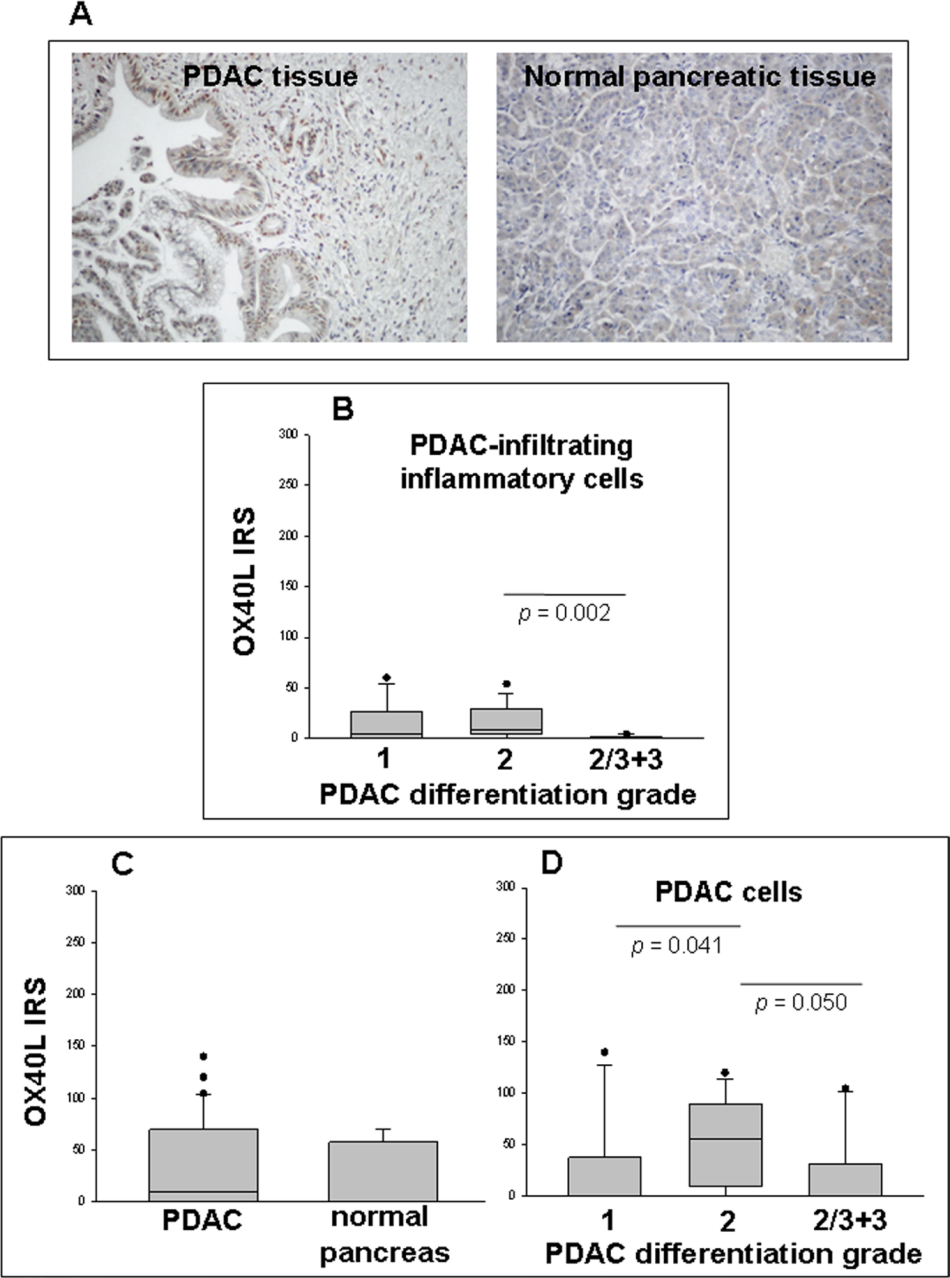
Detection of OX40L in PDAC and normal pancreatic tissues *in situ* as determined by IHC (**A**) Immunostaining of representative samples of PDAC and normal pancreatic tissues (original magnification ×200). (**B**) Immunoreactivity scores (IRS) in inflammatory cell infiltrate of PDAC categorized by tumor differentiation grading. (**C**) IRS in malignant (*n* = 38) and normal (*n* = 8) ductal cells of pancreatic tissue samples. *P*-values obtained by the Mann–Whitney Rank Sum Test. (**D**) IRS in tumor cells of PDAC categorized by tumor differentiation grading. *P*-values obtained by Kruskal–Wallis One Way Analysis of Variance by Ranks followed by Dunn’s Test. Median, 10th, 25th, 75th, and 90th percentiles are presented as vertical boxes with error bars. Dots indicate outliers.

To verify whether TSLP, produced locally by malignant pancreatic ductal cells, was also able to induce infiltration of OX40L^+^ cells *in vivo*, immunohistochemical analysis of OX40L was performed on the same tissue samples analyzed for TSLP expression. Figure 4A shows OX40L immunohistochemical staining of representative specimens of malignant and normal pancreas. OX40L was mainly expressed in cell cytoplasm and nucleus, as has been reported elsewhere [18, 19]. Sixty-one % of PDAC cases displayed tumor-associated inflammatory cell infiltrate, which was not present in normal pancreatic parenchyma. Evaluation of OX40L expression on infiltrating inflammatory cells showed positivity in 41% of the neoplastic tissues analyzed, with an IRS of 4 (0–60) [median (range)]. When the IRS were stratified by disease stage, no statistically-significant difference was found between groups (Supplementary Figure 2A, *p* > 0.05). When PDAC were classified by degree of tumor differentiation (Figure 4B), moderately-differentiated tumors showed higher OX40L expression in infiltrating inflammatory cells than poorly-differentiated tumors [IRS median (range): 9 (0–54) *vs.* 0 (0–4), *p* = 0.002]. By contrast, no significant difference was found between well-differentiated and moderately/poorly-differentiated tumors [IRS median (range): 4.5 (0–60) *vs.* 9 (0–54), *p* = 0.317 and *vs.* 0 (0–4), *p* = 0.112, respectively].

Interestingly, both normal and malignant ductal and acinar cells expressed OX40L (37.5% and 54% of cases, respectively), with no significant difference in IRS terms (*p* = 0.430) (Figure 4C). When the IRS of the positive tumor cases were stratified by disease stage, there was an increasing trend towards the later stages, without achieving statistical significance, likely because of the high inter-individual expression variations [IRS median (range): 0 (0–120), stage I; 9 (0–140), stage II; 75 (0–104.4), stage III + IV] (Supplementary Figure 2B).

If the IRS were subdivided by degree of tumor differentiation, moderately-differentiated tumors appeared to express more OX40L on malignant cells than did well- or poorly-differentiated ones [IRS median (range): 0 (0–140), grade 1; 55 (0–120), grade 2; 0 (0–104.4), grade 2/3 + 3, *p* = 0.041 and *p* = 0.050, respectively)] (Figure 4D). Moreover, a strong positive correlation was found between OX40L IRS of inflammatory cell infiltrate and those of corresponding tumor cells (Spearman correlation test, *r* = 0.536, *p* = 0.0006). Unexpectedly, there were no statistically-significant correlations between TSLP IRS and either inflammatory infiltrate or cancer cell OX40L IRS (Spearman correlation test: *r* = 0.133, *p* = 0.434; and *r* = –0.150, *p* = 0.372, respectively).

### Elevated plasma TSLP levels in patients with locally-advanced or metastatic PDAC

**Figure 5:**
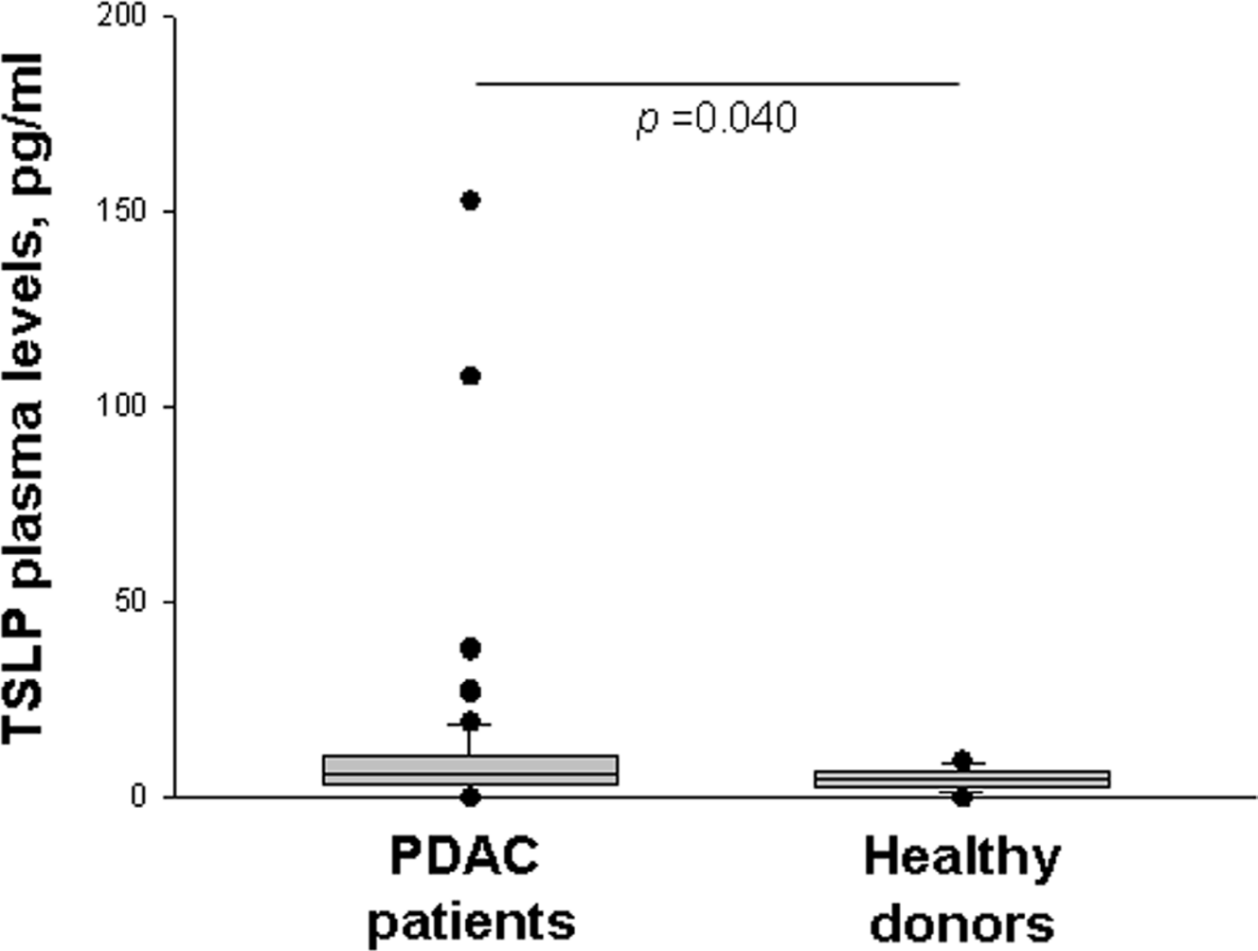
Plasma levels of TSLP Basal circulating TSLP levels in PDAC patients (*n* = 82) and healthy donors (*n* = 41) as determined by ELISA. *P*-values obtained by the Mann-Whitney Rank Sum Test. Median, 10th, 25th, 75th, and 90th percentiles are presented as vertical boxes with error bars. Dots indicate outliers.

To determine whether the tumor microenvironment affects circulating levels of TSLP, basal plasma TSLP concentrations were measured in patients with locally-advanced or metastatic PDAC, and compared with those of normal subjects; the former were higher than the latter [median (range): 5.74 (0–152.9) pg/ml *vs.* 4.65 (0–9.6) pg/ml, *p* = 0.040] (Figure 5).

### Correlation between presence of metastasis and plasma TSLP levels

To clarify the clinical significance of these findings in locally-advanced or metastatic pancreatic cancer, the relationship between presence of metastasis and pre-chemotherapy (time 0) plasma TSLP levels was evaluated. No significant difference and no correlation were detected, by the Mann-Whitney test [median (range) non-metastatic patients’ TSLP at time 0 *vs*. metastatic patients’ TSLP at time 0: 5.74 (0–38.63) pg/ml *vs*. 5.74 (0–152.89) pg/ml; *p* = 0.860] and the Spearman correlation test (*r* = −0.055, *p* = 0.625), respectively.

### Effect of chemotherapy on plasma TSLP

To estimate the possible *in vivo* modulatory influence of chemotherapy on circulating levels of TSLP, its plasma concentration was analyzed comparatively pre- (time 0) and post-treatment at first restaging (time 1) in 62 patients out of 82 enrolled. These patients received one of the following chemotherapy regimens: GEM (*n* = 27), GEMOX (*n* = 32), bolus 5-FU + Calcium Levofolinate (*n* = 2), or BEV+CAPE+RT (*n* = 1). As Table 2 shows, in general pre- and post-chemotherapy circulating TSLP levels did not differ significantly. When the larger groups, i.e. patients treated with GEM or GEMOX were stratified by therapeutic protocol, no difference was found between plasma levels of TSLP pre- and post-chemotherapy in patients treated with GEM alone (*p* = 0.505). Conversely, in the GEMOX group, a statistically-significant increase in TSLP levels was observed at first restaging (*p* = 0.044) (Table 1). Since this increase might be associated to the chemo-resistance that usually occurs in patients with advanced disease, the levels of TSLP in responsive (*n* = 11) *vs*. non-responsive (*n* = 21) PDAC patients under GEMOX treatment were compared. A trend towards higher circulating TSLP level in non-responsive patients was found, but this trend did not reach statistical significance, probably because of the small number of cases and the high inter-individual variation (mean ± SE: 4.96 ± 1.55 *vs.* 18.82 ± 8.58, *p* = 0.257) (Supplementary Figure 3).

**Table 1:** Plasma levels of TSLP pre- and post-treatment

Therapeutic protocol	TSLP, pg/ml		*p* ^e^
	Time 0^a^	Time 1^b^	
Total patients treated (*n* = 62)^c^	4.95 (0–107.9)^d^	4.74 (0–182.06)	0.364
GEM	4.97 (0–14.82)	3.11 (0–16.28)	0.505
GEMOX	4.88 (0–107.91)	4.90 (0–182.06)	0.044

^a^Pre-chemotherapy;

^b^Post-chemotherapy (first restaging).

^c^GEM (*n* = 27), GEMOX (*n* = 32), bolus 5-FU + Levofolinate Calcium (*n* = 2), and BEV+CAPE+RT (*n* = 1).

^d^Median (range) plasma levels measured by ELISA.

^e^Wilcoxon matched pairs test.

**Table 2:** Patients’ clinical and pathological features

Variables	Number of cases (*N* = 82)
**Gender** Female Male	35 (42.7) 47 (57.3)
Age (y)	66.2 ± 9.2
**Disease stage at start of chemotherapy** II A	3 (3.7)
II B III IV	15 (18.3) 13 (15.8) 51 (62.2)
**Surgery** None Palliative Radical	54 (65.8) 5 (6.1) 23 (28.0)
**Metastases** No Yes	31 (37.8) 51 (62.2)
**Metastasis site** Liver Peritoneum Lung Liver, peritoneum	30 (58.8) 6 (11.8) 4 (7.8) 5 (9.8)
Liver, lung Liver, bone Lung, peritoneum Lung, liver, peritoneum Lung, liver, bone	1 (2.0) 1 (2.0) 2 (3.9) 1 (2.0) 1 (2.0)
**Chemotherapy** No Yes Type of chemotherapy GEM GEMOX 5-FU + levofolinate calcium BEV+CAPE+RT	6 (7.3) 76 (92.7)
	34 (44.7) 38 (50.6) 2 (2.6) 2 (2.6)
**Response** Complete remission (CR) Partial remission (PR) Stable disease (SD) Disease progression (DP) Clinical progression	2 (2.6) 4 (5.3) 32 (42.1) 31 (40.8) 7 (9.2)

Age data are presented as the mean ± standard deviation and other data as the number (percentage).

Among patients who passed the first restaging after GEM or GEMOX treatment (*n* = 72), 64.7% of those treated with GEM went into complete remission (*n* = 2, CR), partial remission (*n* = 2, PR), or disease stabilization (*n* = 18, SD), with median (range) survival of 329 (117–1393) days, while 35.3% had disease progression (*n* = 12, DP) with median (range) survival of 160 (43–601) days; among patients treated with GEMOX, 34.2% achieved PR (*n* = 2) or SD (*n* = 11) with median (range) survival of 374 (169–1175) days, while 65.8% had DP (*n* = 25), with a median (range) survival of 148 (80–514) days.

### Objective tumor-response rate and plasma TSLP levels

In 72 treated patients (including 2 patients treated with BEV+CAPE+RT and 2 with bolus 5-FU + Levofolinate calcium) radiologic or clinical response was evaluated. Overall, chemotherapy response-rate was 48.6% (35 responders, comprising 2 CR, 4 PR and 29 SD). 51.4% of patients (*n* = 37) were non-responsive with DP. No statistically-significant correlation between response to chemotherapy and TSLP levels was found (Spearman correlation coefficient: *r* = 0.144, *p* = 0.228).

### Overall survival and plasma TSLP levels

**Figure 6:**
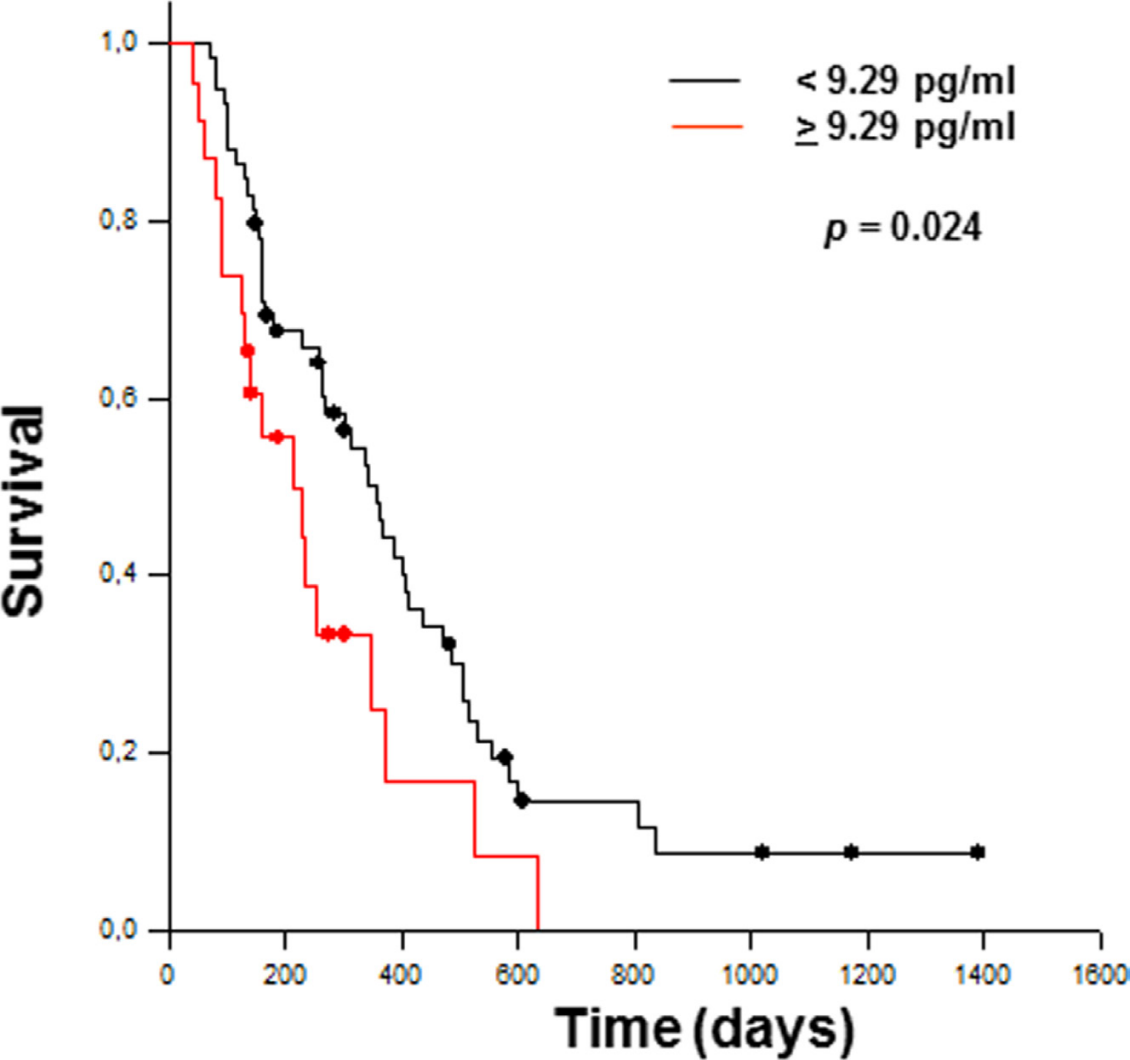
Kaplan–Meier curves estimate overall survival for PDAC patients with <9.29 pg/ml (*n* = 59) and >9.29 pg/ml (*n* = 23) TSLP levels The significant difference between the two groups is shown (Log Rank Test).

Due to the lack of standardized cutoff values for TSLP, the 99th percentile from a healthy reference population (9.29 pg/ml) was taken arbitrarily in this study as cutoff for prognostic assessment. Kaplan-Meier curves for overall patient survival, combined with univariate analysis, showed that patients with high plasma TSLP levels (≥9.29 pg/ml) had significantly shorter overall survival (*p* = 0.024) (Figure 6). Additionally, multivariate analysis with Cox regression model, adjusting for age, gender, radical surgery, metastasis, stage, and chemotherapy (any treatment, computed as dichotomous variable), showed that TSLP levels were statistically independent predictive factors for poorer prognosis for PDAC. [Hazard ratio (HR) = 1.986; 95% confidence interval (CI) 1.012–4.016; *p* = 0.036].

### Diagnostic value of TSLP as a plasmatic biomarker for PDAC

To further evaluate the potential clinical utility of TSLP as diagnostic serum marker, ROC curve analysis was used to discriminate between PDAC patients and healthy volunteers. The cutoff value of 4.76 ng/ml was associated with the highest diagnostic accuracy [*p* = 0.04, Area Under Curve (AUC) = 0.6129, sensitivity 61%, specificity 60%].

## DISCUSSION

This explorative study found that PDAC cells themselves, both *in vitro* and *in situ,* can elaborate TSLP, a cytokine known to promote type 2 activation/maturation of resident tumor DCs. This original finding offers a fresh interpretation of the aberrant deviation of immunity that, in this cancer, may contribute to tumor progression. This study shows that: i) some established pancreatic carcinoma cell lines, e.g. BxPC-3, express both TSLP mRNA and protein; ii) TSLP-rich CM from BxPC-3 cells, in a portion of normal DCs generated *ex vivo*, induce expression of the Th2 polarizing molecule OX40L, partially reversed in the presence of a neutralizing anti-TSLP polyclonal antibody; iii) both tumor infiltrating inflammatory cells and PDAC cells express the OX40L molecule; iv) abnormally elevated TSLP levels may exist *in situ* in tumor cells and, systemically, in locally-advanced/metastatic PDAC patients; v) among patients on different chemotherapeutic protocols, those receiving GEMOX display significantly elevated circulating TSLP levels; vi) circulating TSLP levels show promise as a diagnostic predictor for PDAC; vii) increased plasma TSLP is an independent prognostic risk factor for poor outcome.

It is increasingly clear that TSLP, known to be important in allergy, is also critically involved in the control of tumor growth and metastasis, having different effects depending on context, cellular source, and target [17]. In addition, it has been recently proposed that two isoforms of TSLP, exhibiting distinct or even opposite functions, exist in humans. The constitutive short isoform appears to mediate homeostatic functions, while the inducible long isoform is upregulated during inflammation [20].

The study shows that, of three PDAC cell lines analyzed (Capan-2, PT-45 and BxPC-3), only the latter synthesized relevant levels of TSLP. Interestingly, both *in vitro* study and flow cytometry analysis found that some normal myeloid DCs, generated *ex vivo* in the presence of BxPC-3 cell-CM (but not in that of TSLP-negative Capan-2 cell-CM) express OX40L, a molecule fundamental for optimal induction of primary and memory Th2 responses. However, this effect was only attenuated when the BxPC-3 cell-CM was pre-treated with a neutralizing anti-TSLP polyclonal antibody. This antibody recognizes both short and long TSLP isoforms, suggesting that, in PDAC, TSLP is not the only factor conditioning DCs for Th2 priming. This hypothesis appears reasonable, taking into account that the cytokine IL-18 - one of the most important Th1 cytokines in the presence of IL-12, but that, in its absence, behaves as the Th2 cytokine - induces OX40L in DCs [21]. PDAC cells, both *in vitro* and *in situ*, constitutively express IL-18 [5] which, therefore, in this context may induce DCs to drive Th2 cells, cooperatively with TSLP.

The human tissue microarray study shows that most of the PDAC tissues analyzed expressed TSLP protein at both malignant-cell and stroma levels.. The present study demonstrated that, in addition to CAFs [16], PDAC cells directly produce TSLP *in vivo*, suggesting further immune-response manipulation in promoting tumor growth in the tumor itself.

TSLP elaboration is not solely a PDAC-cell-associated phenomenon. An increasing number of studies have focused on the complicated role of TSLP in several cancers: it is tumor-promoting in some instances, and tumor-inhibiting in others. It has been shown that breast-cancer-derived TSLP, but not tumor-infiltrating fibroblasts, drives tumor-promoting inflammatory Th2 cells present in the malignant microenvironment [22]. In lung cancer tissue, increased levels of TSLP appear to be involved in the marked prevalence of immunosuppressive CD4^+^CD25^+^ T_reg_ cells, which might help tumor cells escape the host immune system [23]. Notably, this finding might explain the concomitant presence of circulating DCs polarized into a tolerogenic phenotype, and the expanded T_reg_ pool found in PDCA patients [4, 6]. Moreover, TSLP produced by cervical cancer cells may promote angiogenesis [24, 25], and down-regulate miR-132 expression, favoring tumor development [26]. The involvement of TSLP in tumor growth is also supported by several murine experimental studies, in which tumor growth was inhibited in TSLP-deficient hosts [27, 28]. Conversely, recent investigations in mouse models, and in early-breast and pancreatic human tumors [29], skin cancer [30, 31] and colon cancer [32], suggest a tumor-suppressing effect for TSLP.

The existence of two isoforms of TSLP controlled by two different promoter regions, and responsible for two opposing biological actions, may explain this paradoxical role of TSLP in malignancies. In the light of this recent finding, since the IHC analysis on PDAC tissue samples reported here did not distinguish between the two TSLP isoforms, and no correlation was found between TLPS protein expression levels and disease stage, the potential roles of the two isoforms in this disease remains to be determined. However, it appears likely that local quantitative and/or qualitative perturbations in the TSLP isoform or TSLP receptor expression may be an initiating and/or driving force, contributing to cancer formation and progression.

To complement the present *in vitro* studies on the contribution of TSLP-rich CM from malignant pancreatic cells to OX40L+ DC generation, IHC analysis on PDAC tissue microarray was performed to determine the presence of the OX40L molecule in cell infiltrate of primary PDAC. Most tumors were infiltrated with OX40L^+^ cells, likely DCs; these, among tumor-infiltrating cells with APC capability, have both the numerical advantage and the broadest range of antigen presentation [33]. Surprisingly, OX40L expression was not restricted to immune-system related cells, but in several cases was also similarly present in both normal and tumor cells.

The role of OX40L in the polarization of Th cells is complex. OX40L is not constitutively expressed, but can be induced on APCs upon CD40 and Toll-like receptor engagement or cytokine signals. TSLP and IL-18 have been shown to potently induce OX40L expression on DCs [14, 34]. The molecule appears to play a universal and essential function in generating Th2 responses [14], whereas it may be connected to a Th1 response, though not always playing a paramount role [35].

OX40L-expressing cells infiltrating the PDAC site may contribute to create inadequate or immunosuppressive events, with the help of OX40^+^ T_reg_ cells accumulating in the tumor tissue; they may also enhance chronic inflammatory responses, promoting tumor invasion and metastasis. Conversely, OX40L^+^ cancer cells can exploit pathways present in normal tissue to establish a microenvironment facilitating tumor development.

Unexpectedly, OX40L expression levels in the PDAC tissue samples were not correlated with those of TSLP, evaluated in parallel. However, this is in line with *in vitro* results, where OX40L-induction on DCs, generated in the presence of BxPC-3 cell-CM, was only partially TSLP-dependent. It provides further evidence that, in PDAC as in allergic inflammation, local TSLP-activated DCs can promote Th2 differentiation in a unique manner (dependent on OX40L and on the lack of production of IL-12), and that IL-18 may cooperatively potentiate the inflammatory Th2 cell responses, furthering tumor progression.

Since TSLP is a secreted protein, and is highly expressed in pancreatic tumor tissues, plasma TSLP levels were measured in a group of locally-advanced/metastatic PDAC patients, and its potential clinical significance was assessed. It emerged, for the first time, that circulating TSLP (irrespectively of its biological activity) was significantly elevated in PDAC patients in comparison with normal subjects, independently of disease stage. The patients enrolled in this systemic analysis had advanced disease. In the light of the results of the *in situ* study performed by IHC on early stage PDAC tissue samples (in prevalence I/II stages), it is tempting to speculate that TSLP release occurs early in PDAC tumorigenesis and, once established, remains constant during the late phase of tumor progression.

This is contrasted by findings reported by Demehri *et al.* in a murine model: TSLP-stimulated CD4^+^ Th2 cells were found to be key mediators of the antitumor immune response that blocks early stages of both breast and pancreatic cancer development, protecting the animals from advanced cancer and metastasis. In particular, in the presence of systemic TSLP, spontaneous pancreatic tumors transferred to K14-TSLP^tg^ mice grew less than those in WT mice, with a significant increase in GATA3^+^ Th2 infiltrating cell numbers [29].

However, these two possibilities are not contradictory, taking into account emerging evidence that TSLP exists in two different isoforms exerting opposing biological effects. It is thus tempting to hypothesize that, in early-stage PDAC, the prevalence of the short TSLP isoform, predominantly produced by cells that are still well/moderately-differentiated, may control tumor growth. But, in response to as-yet-unidentified stimuli altering the local microenvironment, the induced long TSLP isoform then takes over, affecting the course of the disease. At the systemic level, altered circulating levels of TSLP may negatively influence the frequency, composition, and functional activity of DC populations *in vivo*. This assumption is corroborated by *ex vivo* experiments showing that DCs from healthy subjects, generated in the presence of BxPC-3 cell-CM containing TSLP, expressed higher levels of OX40L surface. The mechanisms regulating TSLP expression and secretion from cancer cells, including a potential link with oncogenic events, remain to be established.

Interestingly, univariate and multivariate analysis showed elevated circulating TSLP levels to be significantly associated with poorer survival, and to be an independent indicator of prognosis, respectively. As yet, no directly comparable data are available. However, in WT mice, serum levels of TSLP correlated positively with malignant breast-tumor cells and melanoma growth, indicating the importance of TSLP in tumor progression [27]. Moreover, in gastric cancer, TSLP is closely related to tumor progression and poor survival [36].

In investigating the ability of TSLP to discriminate between patients and controls, ROC analysis showed that the cutoff value of 4.76 ng/ml had the greatest diagnostic accuracy. However, further longitudinal data are required to confirm this observation.

Systemic chemotherapy still remains a standard of care for the treatment of PDAC patients with unresectable locally-advanced and metastatic disease. In this study, among the several therapeutic protocols used, only the double-drug combination GEMOX was found to modulate plasma TSLP levels. These results are in line with research showing that chemotherapeutic agents may have immunomodulating properties and functional interactions [37]. However, unlike GEM monotherapy, which is generally used as adjuvant treatment after radical surgery, the GEMOX regimen is administered in unresectable advanced/metastatic disease. Thus the increase in plasma TSLP levels could be linked to chemo-resistance due to the more aggressive tumor cell behavior, given that the unresponsive patients under GEMOX treatment showed slightly increased circulating levels of TSLP.

Although this patient series is not extensive, when the clinical response to different treatments is evaluated some tentative findings emerge. The clinical response to GEMOX (which is associated to a rise in plasma TSLP levels) was less favorable than GEM alone. However, pretreatment circulating TSLP levels did not predict the response to any regimen.

Taken together, these clinical observations are consistent with the hypothesis that, in advanced-stage PDAC, high local and peripheral TSLP levels could have undesirable effects, producing an immune response that is both inappropriate and persistent. This assumption is highlighted by the importance of TSLP as an independent prognostic factor. Further investigation will thus be needed, in a large cohort of patients with early-stage operable cancer.

The goals of cancer immunotherapy are to activate and expand tumor-specific CD4^+^ and CD8^+^ T cells, as an effective means of augmenting immunity and overcoming mechanisms used by tumors to evade destruction. The results of this study are consistent with the notion that local and systemic tumor-derived TSLP may, with other concomitant immune-suppressive cytokine pathways, contribute to limiting the anti-tumor therapeutic responses to DC-based vaccines in PDAC. However, TSLP’s emerging ambiguous role in tumor immunology complicates the decision over how to manipulate TSLP, or its signaling pathway, in managing cancer patients. Additional studies will be required to reveal the mechanistic basis of the tumor-promoting *vs.* tumor-suppressive effects of TSLP in different cancers, before TSLP can be recognized as a potential target for therapeutic intervention.

## MATERIALS AND METHODS

### Normal and PDAC-established cell lines and preparation of conditioned media (CM)

PDAC-derived cell lines Capan-2, BxPC-3 (ATCC HTB-80 and CRL-1687) and PT-45 (kindly provided by Prof. M. F. Di Renzo, Institute for Cancer Research and Treatment, Candiolo, Italy) were cultured in RPMI 1640 medium supplemented with 10% FCS (Thermo Fisher Scientific, MA, USA) and regularly tested for mycoplasma contamination using Hoechst dye H33258 (Sigma Aldrich, MO, USA). Immortalized human pancreatic ductal epithelial cells HPDE6-E6E7 (H6c7) were generously provided by Dr. Ming-Sound Tsao, Cancer Center/Princess Margaret Hospital, University Health Network, Toronto, Ontario, Canada [38]. CM were obtained as described elsewhere [39].

### Patients

The series comprised 82 patients with locally-advanced or metastatic PDAC admitted to the Department of Medical Oncology, Città della Salute e della Scienza Hospital, Turin, Italy, enrolled into the study between May 2010 and March 2012; patients with serious infections or severe allergic disease were excluded. None of the participants had received chemo/radio therapy before entering the study. All participants signed an informed consent form; study procedures were in accordance with the Declaration of Helsinki. Before starting chemotherapy, patients were staged following the International Union Against Cancer (UICC) TNM system. Patients’ clinical-pathological details are in Table 2. Of the 82 patients, 23 underwent radical surgery followed by adjuvant chemotherapy; 5 received palliative surgical treatment followed immediately by chemotherapy; 54 patients, judged unsuitable for surgery, received palliative chemotherapy. Six patients received no antitumor treatment because of the rapid clinical progression of their disease. Patients were subjected to the following chemotherapeutic protocols: gemcitabine (GEM) (1250 mg/m^2^ on days 1 and 8 every 21 days); GEM (1000 mg/m2 on day 1) plus oxaliplatin (OX) (100 mg/m^2^ on day 2 every 14 days); bevacizumab (5 mg/kg every 14 days) plus capecitabine (825 mg/m^2^/b.i.d.) and concomitant radiotherapy (50.4 Gy in 28 cycles) (BEV+CAPE+RT), following the study protocol; 5-fluorouracil (5-FU) (500 mg/m^2^) plus levofolinate calcium (250 mg/m^2^ on days 1, 8, and 15 every 28 days). After the first restaging, responses (assessed by the RECIST criteria) were as follows: 2 complete remission (CR), 4 partial remission (PR), 32 stable disease (SD) and 31 disease progression (PD) (in 7 cases clinical progression was not documented radiologically).

Patient follow-up continued until death or March 2012. 65 patients died (median survival 242.5 days; range 43–836). 17 patients were alive at completion of the study. Survival was 51.2% at 1 year after diagnosis.

### Cell isolation and generation of DCs

Peripheral blood mononuclear cells (PBMC) were isolated from heparinized blood samples from healthy donors (*n* = 10). *Ex vivo*-generated DCs were obtained as described elsewhere [4], in the presence or absence of 20% Capan-2 and BxPC-3 cell-CMs. In selected experiments, DCs were generated in the presence of 20% BxPC-3 cell-CM, pre-treated or not with neutralizing polyclonal antibody against TSLP (R&D Systems, Minneapolis, MN, 40 ng/ml final dilution), or with an appropriate irrelevant antibody.

### Depletion of TSLP using Protein A/G-coated agarose beads

Neutralizing polyclonal antibody against TSLP (R&D Systems) or irrelevant control IgG were added to BxPC-3 cell-CM at the concentration used to neutralize TSLP biological activity (40 ng/ml) as reported above. Samples were incubated for 30 min at r.t. After addition of Protein A/G agarose beads (Thermo Fisher Scientific) (50 µl/ml per CM, amount exceeding the binding capacity of 250 µg of antibody) the samples were incubated o.n. at 4° C under agitation, and then centrifuged twice at 420 g for 5 min to ensure complete removal of agarose beads.

### Determination of TSLP levels

TSLP was measured in the CM of PDAC cell lines and in plasma from PDAC patients and healthy donors, as well as in Bx-PC-3 cell-CM depleted of TSLP by immunoprecipitation, using an ELISA kit (Thermo Fisher Scientific). Minimum detectable quantity: 8 pg/ml. The manufacturer of the ELISA kit was not able to indicate the TSLP isoform recognized.

### RNA isolation and real time reverse-transcription polymerase chain reaction (RT–PCR)

Total RNA was extracted from Capan-2, BxPC-3 and PT-45 cell lines, and from appropriate positive controls, using TRIzol Reagent (Thermo Fisher Scientific). To remove traces of genomic DNA, total RNAs (1 µg) were treated with DNase I and reverse-transcribed to cDNAs using SuperScript II (Thermo Fisher Scientific).

Real time RT-PCR was performed in duplicate using SYBR Green real time PCR master mix (Bio-Rad, CA, USA) in iCycler iQ system (Bio-Rad). The specific primers used to amplify TLPS and β-actin genes were, respectively: FW: CGTGGTGGGAAGAGTTTA and RW: CAGTTAGTGAAGTCGTAAGT; FW: GCGAGAAGATGACCCAGATC and RW: GGATAGCACAGCCTGGATAG. TSLP RNA expression levels, reported as arbitrary units relative to β-actin, were calculated by the Gene Expression Analysis Real-Time PCR Detection System Ò Software Tool for iCycler iQ (Bio-Rad).

### Flow cytometric analysis

Expression of OX40L (CD252) was analyzed on normal DCs generated in the conditions reported above. The cells were double stained with a phycoerythrin-cyanine 5.1 (PC5)-conjugated anti-HLA-DR monoclonal antibody (mAb) (clone ID: Immu-357, Beckman Coulter, CA, USA) and fluorescein isothiocyanate (FITC) anti-OX40L mAb (clone ID: 159403, R&D Systems). Cells were analyzed by flow cytometry in a Coulter Epics XL Cytometer (Beckman Coulter) using the Expo32 software (Beckman Coulter). Results are expressed as the percentage of OX40L^+^ cells in the fraction of HLA-DR+ cells. Statistical analyses were based on at least 30,000 events gated on the population of interest.

### Immunohistochemistry (IHC)

Commercial tissue microarrays (TMA) containing 38 PDAC tissue samples (*n* = 11 well-differentiated, *n* = 2 moderately-differentiated, *n* = 1 moderately/poorly-differentiated, *n* = 3 poorly-differentiated; from 13 male and 25 female patients, aged 41–74 years, median age 67.5; *n* = 9 disease stage I, *n* = 23 stage II, *n* = 2 stage III, and *n* = 3 stage IV) and 8 healthy pancreatic tissue samples (2 male and 6 female donors, aged 31–41 years, median age 35) (U.S. Biomax, MD, USA) were used to identify and localize TSLP and OX40L proteins by IHC. TMA slides were processed for IHC as described elsewhere [5]. Following incubation with a rabbit mAb against human TSLP (clone ID: EPR4191, Epitomics, CA, USA) and a mouse mAb against human OX40L (clone ID: 15940, R&D Systems), the sections were stained using peroxidase-based visualization DAKO LSAB^®^ kit (Dako, Denmark). Staining intensity was evaluated by a semiquantitative method, using the immunoreacting score (IRS) proposed by Remmele and Stegner [40], in which IRS = SI (staining intensity) x PP (percentage of positive cells). SI was assigned as: 0 = negative; 1 = weak; 2 = moderate; 3 = strong. PP was assigned as: 0 = negative; 1 = 1–20% positive cells; 2 = 21–50% positive cells; 3 = 51–100% positive cells. Ten visual fields from different areas of each specimen were chosen at random for IRS evaluation.

### Statistical analysis

The Mann-Whitney Rank Sum Test, Kruskal-Wallis One Way Analysis of Variance by Ranks or Student’s *t*-test were used to evaluate statistically-significant differences between datasets. Correlations were analyzed by the Spearman Test. Overall patient survival was defined as the time from the date of diagnosis to the date of last follow-up (censored) or patient death from any cause. Survival probabilities were calculated using the Kaplan–Meier Method. Survival curves were compared using the Log-Rank Test (Mantel-Cox). The feasibility of using plasma TSLP as a biomarker for differentiating cases from normal controls was assessed by receiver-operating characteristic (ROC) curve analysis. *P* values < 0.05 were considered statistically significant. Statistical analysis was performed with SPSS Statistics for Windows, Version 22.0 (SPSS Inc, IL, USA).

## SUPPLEMENTARY MATERIALS FIGURES AND TABLE



## Abbreviations

TSLP: Thymic stromal lymphopoietin
OX40L: OX40 Ligand
DC: dendritic cell
PDAC: pancreatic ductal adenocarcinoma
Th: T helper
IL: Interleukin
CAF: cancer-associated fibroblast
CM: conditioned media
RT-PCR: reverse-transcription polymerase chain reaction
IRS: immunoreacting score
BEV: bevacizumab
CAPE: capecitabine
RT: radiotherapy
GEM: gemcitabine
GEMOX: gemcitabine plus oxaliplatin
(CR): complete remission
PR: partial remission
SD: stable disease
PD: disease progression
