# Supplementary material for: Thymic stromal lymphopoietin in human pancreatic ductal adenocarcinoma: expression and prognostic significance
Source: Oncotarget. 2018 Aug 28;9(67):32795–809. doi: 10.18632/oncotarget.25997 (PMC6132354; doi:10.18632/oncotarget.25997)
Supplement: Supplementary file 1 [file oncotarget-09-32795-s001.pdf]

Thymic stromal lymphopoietin in human pancreatic ductal adenocarcinoma: expression and prognostic significance

SUPPLEMENTARY MATERIALS

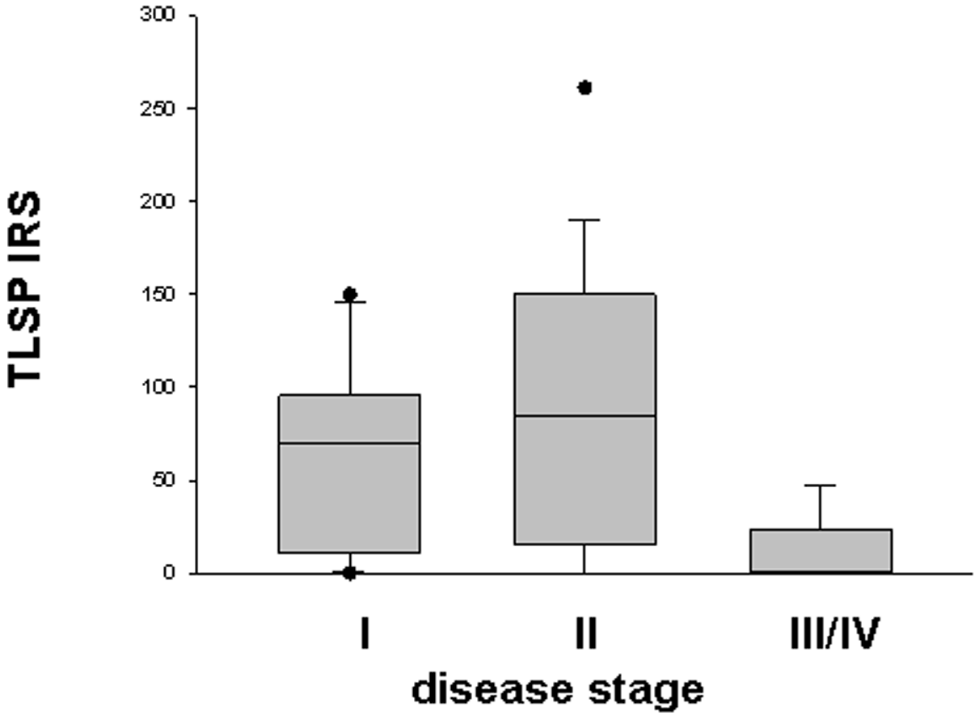

Supplementary Figure 1: TSLP immunoreactivity scores (IRS) in PDAC categorized by disease stage. .No statistical significant difference between the groups was present (*p*-values > 0.05, Kruskal-Wallis One-Way Analysis of Variance by Ranks). Median, 10th, 25th, 75th, and 90th percentiles are presented as vertical boxes with error bars. Dots indicate outliers.

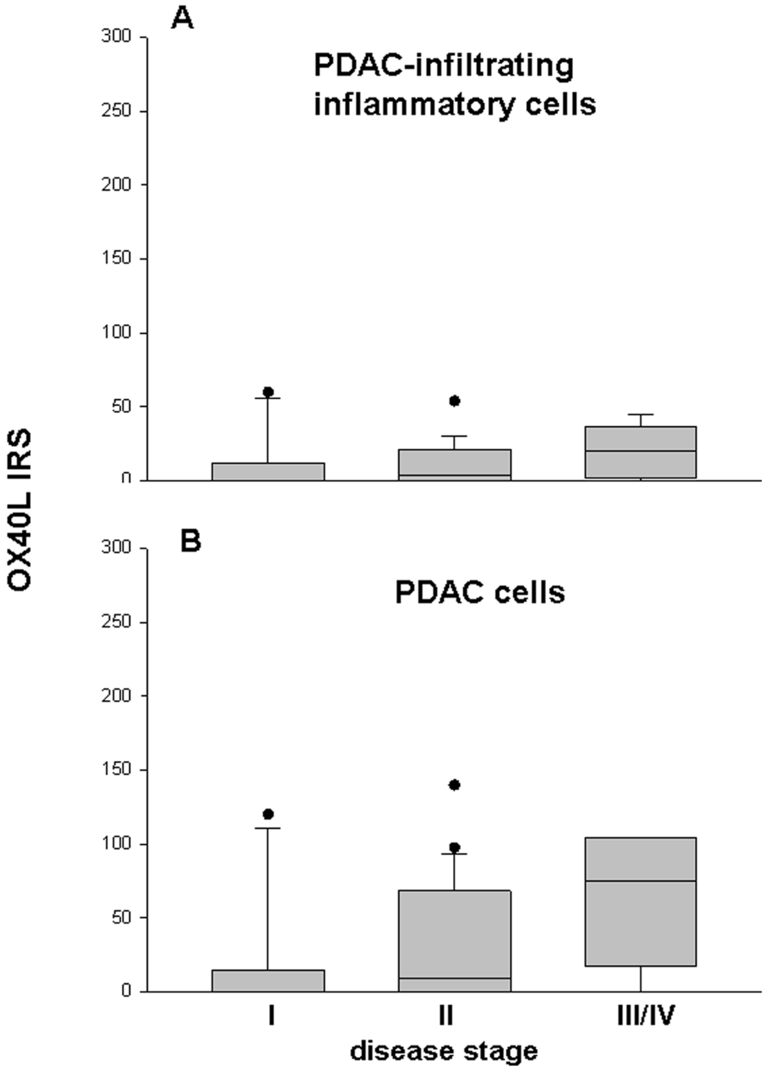

Supplementary Figure 2: OX40L immunoreactivity scores (IRS) in PDAC-inflammatory cell infiltrate (**A**) and PDAC cells (**B**) categorized by disease stage. No statistical significant difference between the groups was present (*p*-values > 0.05, Kruskal-Wallis One-Way Analysis of Variance-by-Ranks). Median, 10th, 25th, 75th, and 90thpercentiles are presented as vertical boxes with error bars. Dots indicate outliers.

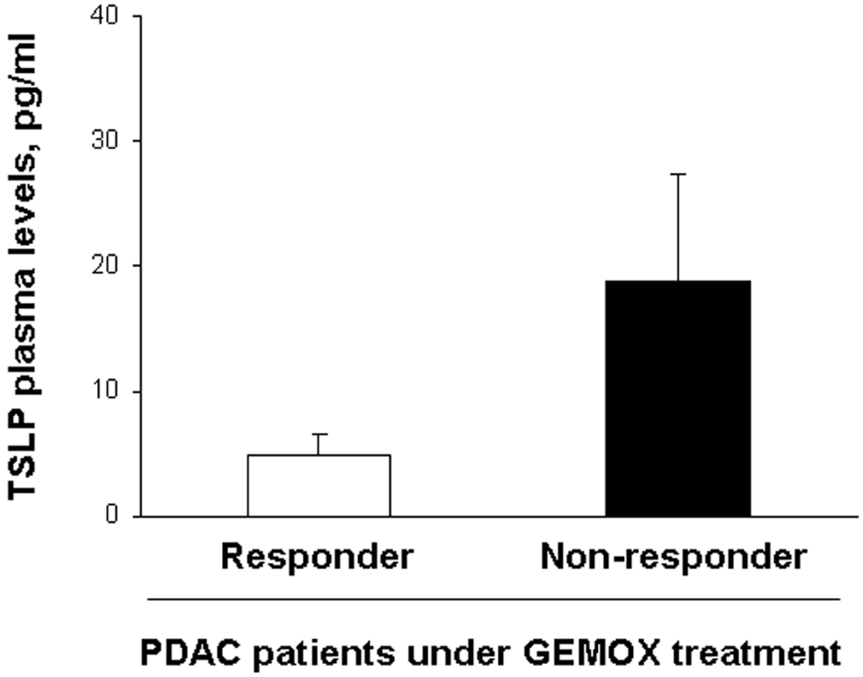

Supplementary Figure 3: TSLP plasma levels in responsive (n = 11) *vs.* non-responsive (n = 21) PDAC patients under GEMOX treatment. No statistical significant difference between the two groups was present (p = 0.257, Student's t-test). Values are means ± SE.

Supplementary Table 1: Depletion of TSLP using protein A/G- coated agarose beads

BxPC-3 cell-CM	BxPC-3cell-CM + irrelevant IgG	BxPC-3 cell-CM + neutralizing anti-TSLP polyclonal antibody
42.21 ± 6.54[a]	39.91 ± 7.66 [a]	n.d. [b]

Levels of TSLP (pg/ml)[a] determined by ELISA in three different BxPC-3 cell-CM before and after immunoprecipitation with irrelevant IgG or the neutralizing anti-TSLP polyclonal antibody bound to Protein A/G-coated agarose beads; [b]n.d = not detectable.
